# Borderline ovarian tumors: clinical characteristics, management, and outcomes - a multicenter study

**DOI:** 10.1186/s13048-016-0276-1

**Published:** 2016-10-18

**Authors:** Mehmet Gokcu, Kemal Gungorduk, Osman Aşıcıoğlu, Nilüfer Çetinkaya, Tayfun Güngör, Gonca Pakay, Zeliha Fırat Cüylan, Tayfun Toptaş, Ramazan Özyurt, Elif Ağaçayak, Aykut Ozdemir, Onur Erol, Anıl Turan, Varol Gülseren, Mehmet Sait İcen, Taylan Şenol, Hakan Güraslan, Burcu Yücesoy, Ahmet Sahbaz, Ozgu Gungorduk, Berhan Besimoğlu, Kaan Pakay, Osman Temizkan, Muzaffer Sancı, Tayup Şimşek, Mehmet Mutlu Meydanlı, Mehmet Harma, Levent Yaşar, Birtan Boran, Aysel Derbent Uysal, Ateş Karateke

**Affiliations:** 1 Department of Gynecology and Gynecologic Oncology, Izmir Tepecik Education and Research Hospital, Izmir, Turkey; 2 Department of Gynecology and Gynecologic Oncology, Mugla Sıtkı Kocman University Education and Research Hospital, Mentese, 48000 Mugla Turkey; 3 Department of Gynecology and Gynecologic Oncology, Sisli Hamidiye Etfal Education and Research Hospital, Istanbul, Turkey; 4 Department of Gynecology and Gynecologic Oncology, Zekai Tahir Burak Education and Research Hospital, Ankara, Turkey; 5 Department of Gynecology and Gynecologic Oncology, Zeynep Kamil Education and Research Hospital, Istanbul, Turkey; 6 Department of Gynecology and Gynecologic Oncology, Akdeniz University School of Medicine, Antalya, Turkey; 7 Department of Gynecology and Gynecologic Oncology, Istanbul Education and Research Hospital, Istanbul, Turkey; 8 Department of Gynecology and Gynecologic Oncology, Dicle University School of Medicine, Diyarbakır, Turkey; 9 Department of Gynecology and Gynecologic Oncology, Bakırköy Dr. Sadi Konuk Education and Research Hospital, Istanbul, Turkey; 10 Department of Gynecology and Gynecologic Oncology, Antalya Education and Research Hospital, Antalya, Turkey; 11 Department of Gynecology and Gynecologic Oncology, Zonguldak Bulent Ecevit University School of Medicine, Zonguldak, Turkey

**Keywords:** Borderline ovarian tumor, Adjuvant chemotherapy, Surgery

## Abstract

**Background:**

The optimal surgical management and staging of borderline ovarian tumors (BOTs) are controversial. Institutions have different surgical approaches for the treatment of BOTs. Here, we performed a retrospective review of clinical characteristics, surgical management and surgical outcomes, and sought to identify variables affecting disease-free survival (DFS) and overall survival (OS) in patients with BOTs.

**Methods:**

A retrospective review of ten gynecological oncology department databases in Turkey was conducted to identify patients diagnosed with BOTs. The effects of type of surgery, age, stage, surgical staging, complete versus incomplete staging, and adjuvant chemotherapy were examined on DFS and OS.

**Results:**

In total, 733 patients with BOTs were included in the analysis. Most of the staged cases were in stage IA (70.4 %). In total, 345 patients underwent conservative surgeries. Recurrence rates were similar between the conservative and radical surgery groups (10.5 % vs. 8.7 %). Furthermore we did not find any difference between DFS (HR = 0.96; 95 % confidence interval, CI = 0.7–1.2; *p* = 0.576) or OS (HR = 0.9; 95 % CI = 0.8–1.1; *p* = 0.328) between patients who underwent conservative versus radical surgeries. There was also no difference in DFS (HR = 0.74; 95 % CI = 0.8–1.1; *p* = 0.080) or OS (HR = 0.8; 95 % CI = 0.7–1.0; *p* = 0.091) between complete, incomplete, and unstaged patients. Furthermore, receiving adjuvant chemotherapy (CT) for tumor stage ≥ IC was not an independent prognostic factor for DFS or OS.

**Conclusions:**

Patients undergoing conservative surgery did not show higher recurrence rates; furthermore, survival time was not shortened. Detailed surgical staging, including lymph node sampling or dissection, appendectomy, and hysterectomy, were not beneficial in the surgical management oF BOTs.

## Background

Taylor first described a type of ovarian tumor in 1929 that was different from both benign and malignant epithelial ovarian tumors [1]. Then, in 1973, the World Health Organization (WHO) assigned the name ‘borderline’ to these tumors, with morphological criteria (especially the absence of stromal invasion) [2]. Borderline ovarian tumors (BOTs) are currently staged according to the International Federation of Gynecology and Obstetrics (FIGO) classification of ovarian cancer; they represent ~10–20 % of all ovarian neoplasias [3]. They are diagnosed in younger women, at an earlier stage, and have a better prognosis than malignant ovarian tumors [4]. The 5-year survival rates are 95–97 % and ~70 % of these tumors are stage I at the time of diagnosis [3].

Preoperative diagnosis of BOTs remains difficult [5]. Cancer antigen 125 (CA-125) is the most helpful available marker in the diagnosis of advanced-stage cases [6]. Moreover, the optimal surgical management and staging of BOTs are controversial. Institutions have different surgical approaches for the treatment of BOTs. Some surgeons prefer comprehensive surgical staging, including lymphatic sampling or dissection, while others exclude the lymph nodes [4, 7].

The purposes of the present study were to perform a retrospective review of the clinical characteristics, surgical management, and surgical outcomes, and to identify variables affecting survival in 733 patients with BOTs who were treated at ten gynecology departments in Turkey. We also sought to explore the following issues:

1. Is surgical staging (including lymphadenectomy) necessary in all patients?
2. Is appendectomy necessary in patients with mucinous BOTs?
3. Which type of procedure should the surgeon choose in patients with BOTs (radical vs. conservative)?
4. Should patients with BOTs receive adjuvant chemotherapy after the surgery if they have a tumor stage ≥ IC, as with epithelial ovarian tumors?
5. What is the function of analyzing frozen sections during BOT surgery? Do the results differ between serous and mucinous types?
6. What should the surgeon do in patients with recurrent BOTs?
7. What are the prognostic factors for overall and disease-free survival in patients with BOTs?

## Methods

This retrospective study was performed using 10 gynecological oncology department databases (Izmir Tepecik Education and Research Hospital, Antalya Akdeniz University School of Medicine, Dicle University School of Medicine, Zekai Tahir Burak Education and Research Hospital, Zonguldak Bulent Ecevit University School of Medicine, Antalya Education and Research Hospital, Zeynep Kamil Education and Research Hospital, Istanbul Bakırköy Sadi Konuk Education and Research Hospital, Sisli Hamidiye Etfal Education and Research Hospital, and Istanbul Education and Research Hospital). All patients with BOTs diagnosed between January 1, 1998 and December 31, 2014 were included.

This study was approved by the ethics committee. It was conducted in accordance with the ethical standards of the Declaration of Helsinki.

From the hospital databases, patient age, menopausal state, pre-operative CA-125, and preoperative ultrasound images were collected. Furthermore, the surgical technique, histological type, mean tumor diameter, lymph node status, stage at diagnosis, final pathological diagnosis, and accompanying pathologies, if any, were reviewed. Additionally, chemotherapy after surgery, postoperative follow-up periods, and data related to disease recurrence were evaluated. If frozen sections (FS) were analyzed intraoperatively, FS results were reported intraoperatively as benign, borderline tumor, at least borderline tumor, or malignant tumor. Patients with incomplete data were excluded from the analysis.

The International Federation of Gynecology and Obstetrics (FIGO) 2009 staging scheme for epithelial ovarian carcinomas was used in all patients [8]. Although the FIGO ovarian staging classification was revised on January 1, 2014, we used the previous staging classification for 2014 patients for consistency. Surgical procedures were classified as radical or conservative. If both ovaries were removed, this was included in the radical group. The conservative group included fertility sparing surgeries (such as unilateral salpingo-oophorectomy (USO), cystectomy, bilateral cystectomy, cystectomy with contralateral ovarian biopsy, and bilateral ovarian biopsies) in women who were premenopausal or wished to preserve their fertility. Moreover, patient operations were categorized into three groups: complete staging, incomplete staging, or unstaged procedures. Complete staging was defined as peritoneal washing and/or biopsies, pelvic and paraaortic lymphadenectomy (sampling or complete), and omentectomy being performed. If only peritoneal washing and omental and/or peritoneal biopsies without lymphadenectomy were performed, this was considered incomplete staging. Furthermore, if only ovarian surgery (only ovarian cystectomy or oopherectomy) was performed, this was considered unstaged. Additionally, if the patients underwent only an appendectomy with ovarian surgery, they were classified in the unstaged group.

Survival analysis was based on the Kaplan-Meier method and results were compared using the log-rank test. Disease-free survival (DFS) was defined as the time from the date of primary surgery to the detection of recurrence or the latest observation. Overall survival (OS) was defined as the time from the date of primary surgery to death or the latest observation. The χ^2^ test and Student’s t-test for unpaired data were used for statistical analyses. Cox regression analysis was used to determine factors affecting survival, and results are presented as hazard ratios (HR). All statistical analyses were performed using the Med-Calc software (ver. 11.5 for Windows; MedCalc Software, Mariakerke, Belgium). A *p* value < 0.05 was considered to indicate statistical significance.

## Results

We evaluated 733 patients with BOTs during the study period. The characteristics of the patients are shown in Table 1. There was recurrence in 69 (9.4 %) of the patients: 35 (50.7 %) in the conservative surgery group and 34 (49.3 %) in the radical surgery group (the difference was not statistically significant, *p* = 0.405) (Table 2). Furthermore no statistically significant difference in recurrence was observed between complete, incomplete, and unstaged patients (9.6, 12.3, and 8.4 %, respectively; *p* = 0.615). Most of the recurrent patients were treated with surgery; 10 were managed with chemotherapy, 47 were managed with surgery, and 12 were managed with chemotherapy after surgery. During the follow-up period, in total, 10 (1.4 %) patients died from their disease.

**Table 1:** Demographic characteristics of patients with borderline ovarian tumors

Age	
Median (*n*, range in years)	41.2 (16–82)
< 40 years (*n*, %)	353 (48.2)
≥ 40 years (*n*, %)	380 (51.8)
Postmenopausal status (*n*, %)	227 (31.0)
Histology	
Serous (*n*, %)	534 (72.9)
Mucinous (*n*, %)	160 (21.8)
Other (*n*, %)	39 (5.3)
Ultrasound image	
Solid (*n*, %)	110 (15.0)
Cystic (*n*, %)	130 (17.7)
Unknown (*n*, %)	493 (67.3)
Median CA-125 level (U/mL)	89 (1–3394)
Median size (mm)	112.02 ± 64.57
Stage in diagnosis (*n*, %)	
IA	516 (70.4)
IB	74 (10.1)
IC	64 (8.7)
IIA	2 (0.3)
IIB	6 (0.8)
IIC	8 (1.1)
IIIA	0
IIIB	11 (1.5)
IIIC	52 (7.1)
IV	0
Disease-free survival (*n*, range in months)	51.7 (1–216)
Overall survival (*n*, range in months)	55.2 (1–216)
Duration of follow-up (*n*, range in months)	55.2 (1–216)

**Table 2:** Pathological and surgical characteristics of patients with borderline ovarian tumors

Frozen pathology records (*n*, %)	
Benign	30 (7.3)
Borderline	251 (61.6)
At least borderline	117 (28.7)
Malignant	9 (2.2)
Accuracy of frozen pathology (*n*, %)*	
Serous (*n*, %)	288 (94)
Mucinous (*n*, %)	67 (80)
Surgery Type (*n*, %)	
Conservative	345 (47.1)
Radical	388 (52.9)
Conservative surgery type (*n*, %)	
Unilateral cystectomy	106 (30.7)
Bilateral cystectomy	20 (5.7)
Cystectomy and contralateral ovarian biopsy	7 (2.0)
Unilateral salpingo-oopherectomy (USO)	194 (56.2)
USO and contralateral ovarian biopsy	17 (4.9)
Bilateral ovarian biopsy	1 (0.2)
Staging surgery (*n*, %)	
None	273 (37.2)
Yes	
Complete	395 (53.9)
Incomplete	65 (8.9)
Received postoperative chemotherapy	101 (13.8)
Appendectomy (*n*, %)	289 (39.4)
Appendectomy with serous histology	159
Appendectomy with mucinous histology	130
Appendicial involvement (*n*, %)	23 (3.1)
Appendicial involvement + mucinous type	21 (2.8)
Hysterectomy (*n*, %)	436 (59.5)
Median removed lymph nodes (*n*, range in numbers)	25.7 ± 22.2 (2–173)
Surgery type (*n*, %)	
Laparascopy	34 (4.6)
Laparotomy	699 (95.4)
Recurrence (*n*, %)	69 (9.4)
Treatment after recurrence**	
Surgery	47 (6.4)
Chemotherapy	10 (1.4)
Surgery + Chemotherapy	12 (1.6)
Recurrence (*n*, %)***	
Conservative group	35 (10.5)
Radical group	34 (8.7)
Recurrence (*n*, %)****	
Unstaging	23 (8.4)***
Incomplete staging	8 (12.3)
Complete staging	38 (9.6)

*P < 0.001

**P < 0.001

***P = 0.405

****P = 0.615

In 407 patients, frozen section analyses were carried out. Benign, borderline, at least borderline, and malignancies were seen in 30 (7.3 %), 251 (61.6 %), 117 (16.0 %), and 9 (2.2 %) patients, respectively. The accuracy of frozen section analyses in serous type tumors was significantly higher than in the mucinous type (94 % vs. 80 %; *p* < 0.001).

In total, 101 patients received adjuvant chemotherapy (CT). During the study period, postoperative CT was administered for FIGO stage IC and more advanced stages or recurrent disease. Of the 101 patients, 25 received CT for recurrent disease, 36 for early stage disease (stage IC or II, 36/76 patients), and 40 received CT for advanced-stage disease (stage III or IV, 40/76 patients). Postoperative CT regimens consisted of cisplatin + paclitaxel (37/101 patients), carboplatin + paclitaxel (55/101 patients), cisplatin + doxorubicin + cyclophosphamide (4/101 patients), cisplatin + cyclophosphamide (3/101 patients), and cisplatin + amifostin (2/101 patients) for 3–6 cycles.

Surgical characteristics of the patients are shown in Table 2. In total, 388 (52.9 %) patients underwent radical excision procedures, while 345 (47.1 %) underwent conservative surgical procedures. An appendectomy was performed in 289 (38.4 %) cases. The number of patients with appendicial involvement was 23 (3.1 %); 21 of them were in the mucinous group (2.8 %).

We next analyzed the patients by dividing them into two groups according to the median age (<40 vs. ≥ 40). All parameters were similar between the groups (Table 3). The results of the multivariate analyses of DFS and OS are shown in Table 4. In the multivariate analysis, performance of surgical staging (or not), FIGO stage, age (<40 or ≥ 40), menopausal status, presence of an invasive implant, performance of radical surgery, lymphadenectomy, and adjuvant CT for tumor stage ≥ IC were not independent prognostic factors for DFS or OS (Table 4).

**Table 3:** Clinical details of patients based on age

	Age < 40 (*n* = 353)	Age ≥ 40 (*n* = 380)	*P* value	RR (95 % CI)
Serous histology^a^	262 (74.1)	272 (71.6)	0.723	
Complete surgery^a^	189 (53.5)	206 (54.2)	0.448	
Radical surgery^a^	184 (52.1)	204 (53.7)	0.673	1.0 (0.7–1.4)
Appendectomy^a^	145 (41.1)	144 (37.9)	0.378	1.0 (0.9–1.2)
Stage I^a^	311 (88.1)	343 (90.2)	0.782	
OS^b^	57.7 ± 42.1	52.9 ± 43.4	0.419	
DFS^b^	53.5 ± 39.7	50.0 ± 40.7	0.390	
Recurrence^a^	33 (9.3)	36 (9.5)	0.954	0.9 (0.6–1.5)

*OS* overall survival, *DFS* disease-free survival

Data are expressed as ^a^: *n* (%), ^b^: mean ± standard deviation

**Table 4:** Results of multivariate analyses of disease-free survival and overall survival

	Disease-free survival			Overall survival		
	Hazard ratio	95 % CI	*P* value	Hazard ratio	95 % CI	*P* value
Age (<40 vs. ≥ 40)	1.0	0.8–1.2	0.67	1.0	0.8–1.2	0.61
Stage (I/II vs. III/IV)	0.9	0.9–1.0	0.39	0.9	0.9–1.0	0.86
Radical surgery	1.0	0.9–1.0	0.25	1.0	0.9–1.0	0.64
Staging surgery	1.0	0.9–1.0	0.10	1.0	0.9–1.1	0.052
Menopause	0.9	0.8–1.2	0.95	1.0	0.8–1.3	0.57
Invasive implant	0.8	0.6–1.2	0.47	0.8	0.6–1.2	0.51
Lymph node dissection	0.9	0.9–1.1	0.08	0.8	0.7–1.1	0.08
Adjuvant chemotherapy (tumorstage ≥ IC)	0.8	0.6–1.0	0.06	0.9	0.8–1.1	0.07

*CI* confidence interval

With a Kaplan-Meier analysis, we did not find any difference in DFS (HR = 0.96; 95 % CI = 0.7–1.2; *p* = 0.576) or OS (HR = 0.9; 95 % CI = 0.8–1.1; *p* = 0.328) between patients who underwent conservative versus radical surgeries. There was also no difference in DFS (HR = 0.74; 95 % CI = 0.8–1.1; *p* = 0.080) or OS (HR = 0.8; 95 % CI = 0.7–1.0; *p* = 0.091) between complete, incomplete, and unstaged patients. When the impact of lymph node sampling or dissection on DFS and OS was assessed, lymph node removal had no effect on DFS (HR = 1.2; 95 % CI = 0.9–1.4; *p* = 0.465) or OS (HR = 1.1; 95 % CI = 1.0–1.3; *p* = 0.623). Furthermore, there was no difference in DFS (HR = 1.3; 95 % CI = 1.1–1.5; *p* = 0.410) or OS (HR = 0.8; 95 % CI = 0.6–1.1; *p* = 0.856) between patients treated with surgery alone, chemotherapy alone, or sequential treatment for recurrent BOT. Similarly, in patients who underwent cystectomies or oopherectomies, there was no difference in DFS (HR = 0.8; 95 % CI = 0.7–1.1; *p* = 0,132) or OS (HR = 0.7; 95 % CI = 0.6–0.8; *p* = 0.212). In BOTs, adding an appendectomy to the surgical procedure had no effect on DFS (HR = 0.9; 95 % CI = 0.7–1.1; *p* = 0.270) or OS (HR = 1.0; 95 % CI = 0.7–1.3; *p* = 0.320). In patients who underwent hysterectomies (vs. not), there was no also no difference in DFS (HR = 1.1; 95 % CI = 0.9–1.2; *p* = 0.208) or OS (HR = 1.4; 95 % CI = 1.1–1.7; *p* = 0,416). Finally, performing (vs. not) an appendectomy in mucinous BOT patients had no effect on DFS (HR = 0.9; 95 % CI = 0.7–1.3; *p* = 0.990) or OS (HR = 0.8; 95 % CI = 0.6–0.9; *p* = 0.751).

We also analyzed the patients by dividing them into two groups according to recurrence (vs. not). All parameters were similar between groups (Table 5). The results of univariate and multivariate analyses for recurrence are shown in Table 5. In univariate and multivariate analyses, age (<40 vs. ≥ 40), FIGO stage (≥ IC vs. < IC), performance of radical surgery, and performance of surgical staging (vs. not) were not independent risk factors for the recurrence of BOTs (Table 6).

**Table 5:** Characteristics of patients based on recurrence

	Recurrence (*n* = 69)	No recurrence (*n* = 664)	*P* value	RR (95 % CI)
Age (years)^a^	41.5 ± 13.8	39.0 ± 12.4	0.146	
Tumor size (mm)^a^	113.5 ± 65.1	93.5 ± 53.8	0.094	
Surgery type^b^			0.470	0.6 (0.1–2.4)
Laparascopy	2 (5.9)	32 (94.1)		
Laparatomy	67 (9.6)	632 (90.4)		
Stage^b^			0.129	0.6 (0.4–1.1)
≥ IC	18 (12.6)	125 (87.4)		
< IC	50 (8.5)	540 (91.5)		
Radical Surgery^b^			0.405	1.2 (0.7–1.9)
Yes	34 (8.8)	354 (91.2)		
No	35 (10.2)	310 (89.8)		

Data are expressed as ^a^: mean ± standard deviation,^b^: *n* (%)

**Table 6:** Results of univariate and multivariate analyses of risk factors of patients with recurrent BOTs

	Univariate analysis			Multivariate analysis		
	Hazard ratio	95 % CI	*P* value	Hazard ratio	95 % CI	*P* value
Age (years) (<40 vs. ≥ 40)	1.0	0.6–1.7	0.845	1.1	0.6–1.8	0.877
Stage (< IC vs. ≥IC)	0.9	0.8–1.0	0.314	0.8	0.7–0.9	0.322
Radical surgery	1.0	0.9–1.1	0.968	1.0	0.9–1.1	0.808
Staging surgery	0.9	0.7–1.2	0.541	0.9	0.6–1.2	0.519

## Discussion

In this study, we performed a retrospective analysis of 733 patients with BOTs who were treated with surgery at 10 gynecology centers in Turkey. BOTs are classified as a separate entity within ovarian malignancies because of their atypical properties. Furthermore, they are not a rare clinical entity, constituting ~10–20 % of all ovarian neoplasias in clinical studies [3]. The present study is one of the largest reported series of cases with BOTs. Similar to previous studies, the mean age of patients with BOTs was 41.2 years in our study. Of the 733 patients with the tumor, 353 (48.1 %) were < 40 years old; our result adds to current knowledge on the occurrence of BOTs in younger and premenopausal women [9–11]. Furthermore, we found that clinical features were similar between the groups aged under and over 40 years. Consistent with previous studies [11, 12], the most common histological type was a serous BOT in the present study. However, the rate of serous histology (72.9 %) was significantly higher than in several previous studies [13, 14]. Heterogeneity of the mucinous tumors may be a reason for this.

The most commonly discussed questions about BOTs are as follows. We address each in turn.

1. Is surgical staging (including lymphadenectomy) necessary in all patients?
  In our study, more than half of our patients were staged surgically, and most of them were staged completely, similar to the ovarian cancer surgery situation. However, we found no difference between the survival rates of staged and unstaged patients. Furthermore, no difference was found between completely and incompletely staged patients. These results were similar to previous studies [13–15]. We found that lymph node removal in surgical staging did not affect survival, as in Güvenal et al.’s study [13]. Similarly, Fauvet et al. suggested that lymph node removal is not a part of surgical staging for BOTs [7].
2. Is appendectomy necessary in patients with mucinous BOTs?
  Adding an appendectomy to surgical staging procedures has been recommended for mucinous tumors, in particular [4]. In our study, ~40 % of cases underwent appendectomies. We found that an appendectomy had no impact on survival in mucinous or other types of BOT. Thus, it is not necessary to perform an appendectomy routinely in patients with mucinous BOTs, according to our findings. Kleppe et al.’s and Lin et al.’s studies reached the same conclusion [16, 17].
3. Which type of procedure (radical vs. conservative) should the surgeon choose in patients with BOTs?
  There is an important and controversial issue about surgical approaches in diagnosed BOT patients, especially in women who wish to preserve their reproductive status. Many previous studies [13, 18] have suggested that patients who undergo conservative surgery have higher recurrence rates than the radical surgery group. Güvenal et al.’s study showed that patients who underwent radical surgery had a 3 % recurrence rate, whereas in patients who underwent fertility sparing surgeries, the recurrence rate was 8.3 % [13]. Furthermore, Boran et al. reported that patients who underwent radical surgery had no recurrence, whereas in patients who underwent conservative surgery, the recurrence rate was 6.5 % [18]. In contrast, we found that the rate of recurrence between the two groups was not different (35/345, 10.5 % vs. 34/388, 8.7 %; *p* = 0.405). Furthermore, we found that radical surgery was not an independent prognostic factor for DFS or OS. Thus, we considered that performing radical surgery makes no sense with regard to recurrence in BOT patients. We attribute these findings to the follow-up period of our study, which was much longer than those of other studies (e.g., Boran et al. and Güvenal et al.). Ayhan et al., suggested that patients with BOTs can be treated safely with conservative surgery [11]. In the present study, we found that surgical procedure (radical vs. conservative) was not an independent prognostic factor for DFS or OS. These findings were similar to those of previous studies [13, 18, 19]. We also demonstrated that hysterectomy had no impact on survival in BOT patients, similar to Menczer et al.’s study [20].
4. Should patients with BOTs receive adjuvant chemotherapy after surgery if they have a tumor of stage ≥ IC, as with epithelial ovarian tumors?
  Based on the literature, the use of adjuvant chemotherapy for BOTs remains controversial. According to the National Comprehensive Cancer Network (NCCN), the treatment recommendation after comprehensive staging depends on the presence or absence of invasive implants. The initial therapeutic approach in patients having invasive implants may include observation; alternatively, consideration can be given to treating patients according to the guidelines for epithelial ovarian cancer (category 2B for adjuvant chemotherapy) [21].
  In the present study, surgery followed by chemotherapy did not show a different survival rate compared to no adjuvant chemotherapy in advanced-stage BOTs. This finding is similar to that of Trope et al. [22]. Most of the recurrence patients were treated with surgery alone in our study, but we found no significant difference in DFS or OS in recurrent patients treated with surgery alone, chemotherapy alone, or sequential treatment.
5. What is the role of frozen section analyses during BOT surgery? Do the results differ between serous and mucinous BOTs?
  An accurate intraoperative diagnosis is important in the management of BOTs during the intra- and postoperative periods. Reported accuracy rates have varied widely, from 50 to 85 %. In this study, the accuracy of frozen section analysis was 90.4 %, slightly higher than many previous studies [23, 24]. One reason may be that there were fewer mucinous tumors in the present study, because mucinous histology has been reported to be associated with low sensitivity in frozen section analyses in past studies [25, 26]. Similarly, in the present study, the accuracy for serous tumors was 94 % versus 80 % for mucinous tumors (*p* < 0.001).
6. What should the surgeon do for patients with recurrent BOTs?
  We found no significant difference in DFS or OS rates in recurrent patients managed with secondary surgery, chemotherapy, or sequential treatment (HR = 1.3; 95 % CI = 1.1–1.5; *p* = 0.410 and HR = 0.8; 95 % CI = 0.6–1.1; *p* = 0.856, respectively). Furthermore we showed that age (<40 vs. ≥ 40), FIGO stage (< IC vs. ≥ IC), performance of radical surgery, and performance of surgical staging (vs. not) were not independent risk factors for recurrence of BOTs. In contrast, Ren et al. reported that a conservative surgical procedure was an independent risk factor for recurrence. Additionally, Sumin et al. reported that age was an independent risk factor for recurrence [12, 27]. One reason for these differences may be that our study included more participants than the other studies.
7. What are the prognostic factors for overall and disease-free survival in BOTs?
  We found that surgical staging (vs. not), FIGO stage, age (<40 vs. ≥ 40), menopausal status, the presence of an invasive implant, radical (vs. conservative) surgery, lymph node dissection (vs. not), and undergoing adjuvant CT for a tumor of stage ≥ IC were not independent prognostic factors for DFS or OS. Our results are similar to many previous studies [13, 14, 28]. In addition to our findings, Güvenal et al. suggested that appendectomy was not an independent prognostic factor for DFS or OS.

This study has several limitations. First, it was a retrospective analysis of patients from various institutions. Second, there were many different clinical approaches. Third, the absence of some data and the histopathological evaluations of BOTs may vary depending on the experience of the institutions. Despite these limitations, this study represents one of the largest series of cases with BOTs, as a 10-center study. Moreover, the availability of good follow-up data increased the validity of the results and mitigated the weaknesses.

## Conclusions

In conclusion, patients undergoing conservative surgeries did not have higher recurrence rates, and survival time was not shortened. Detailed surgical staging, including lymph node sampling or dissection, appendectomy, and hysterectomy did not cause any difference in survival rates. Age and radical surgery were not independent prognostic factors for DFS. Thus, our findings suggest that radical surgery and comprehensive surgical staging should not be routinely performed in BOT patients. We believe that this study shows important findings due to its multi-centric and long-term nature. Although this study was a retrospective analysis, we believe that it provides useful information for prospective randomized controlled trials in the future.

## Abbreviations

BOTs: Borderline ovarian tumors
FIGO: International Federation of Gynecology and Obstetrics
